# Unlocking the Depths: Use of a Trait‐Based Approach to Reveal the Diversity of Foraging Strategies in a Deep‐Pelagic Fish Community

**DOI:** 10.1002/ece3.71891

**Published:** 2025-07-28

**Authors:** Liz Loutrage, Anik Brind'Amour, Benoit Simon‐Bouhet, Rachel Dubourg, Célina Chantre, Jérôme Spitz

**Affiliations:** ^1^ Observatoire Pelagis UAR 3462, La Rochelle Université ‐ CNRS La Rochelle France; ^2^ DECOD (Ecosystem Dynamics and Sustainability) Institut Agro, Ifremer, INRAE Nantes France; ^3^ Centre d'Études Biologiques de Chizé (CEBC) UMR 7372, La Rochelle Université ‐ CNRS Villiers‐en‐Bois France

**Keywords:** bathypelagic, environmental filtering, food acquisition, functional rarity, limiting similarity, mesopelagic

## Abstract

Community trait structure is shaped by environmental filtering and limiting similarity, balancing abiotic selection and competitive divergence. In the context of environmental change and anthropogenic pressures, increasing our understanding of the relative importance of these mechanisms is essential for predicting future changes in biodiversity. The deep‐pelagic ecosystem is characterised by pronounced environmental gradients, particularly in light and food availability. The mechanisms by which fish have adapted to these gradients remain poorly understood. To better understand community trait structure, we measured 26 traits related to foraging function in 42 epi‐ to bathypelagic fish species sampled between 20 and 2000 m depth at night in the Bay of Biscay. We first tested whether limiting similarity or environmental filtering dominates trait structure along the depth gradient by calculating the standardised effect size of four functional diversity indices. Differences in morphological trait values along the depth gradient were then examined using the community weight mean. Species in the epipelagic layer exhibited significant trait convergence, while species in the bathypelagic layer exhibited high functional trait diversity. High locomotor capacity may have been selected in the epipelagic layer, where light and food resources are higher, which could explain the pressure to possess traits adapted for a prey‐tracking strategy. In the bathypelagic layer, species may have to limit the competitive pressure for the food‐scarce conditions by displaying a higher diversity of feeding strategies. The increase in functional space at depth was supported by a few species with a unique combination of traits that may reflect an ambush hunting strategy. The relatively stable environmental conditions at depth may have favoured high functional diversity and rarity. These results raise concerns about the vulnerability of this community to future climate change and potential exploitation, as rare species may provide irreplaceable functions in ecosystems.

## Graphical abstract

The deep‐pelagic fish community showed differences in functional strategies along the depth gradient. Epipelagic species exhibited significant trait convergence with traits adapted to the prey‐tracking strategy, while bathypelagic species exhibited significantly higher functional richness values than expected at random. High functional rarity was observed at depth, with species exhibiting a unique combination of traits that may represent irreplaceable functions within the ecosystem.

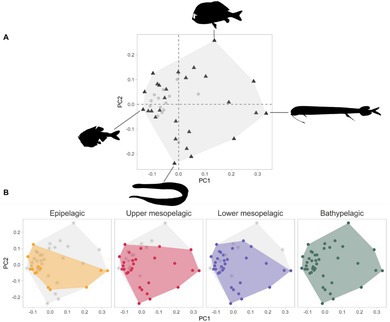

## 1 Introduction

From an evolutionary perspective, the coexistence of species is facilitated by the differentiation of their ecological niches, which enables them to mitigate interspecific competition, particularly for food resources (i.e., the limiting similarity hypothesis) (Gause 1932; Chesson 2000; Rodríguez and Ojeda 2014). The differentiation of species niches can be achieved by the specialisation of certain traits under selective pressure, including morphological, phenological, physiological or behavioural characteristics that can be measured at the individual level (MacArthur and Levins 1967; Violle et al. 2007; Losos 2010; De Bello et al. 2021). Feeding strategies, which are based on specific sets of traits, play a key role in this process. These strategies, by allowing species to access a particular range of food, enable the development and reinforcement of these specialised traits (Brown and Wilson 1956; Rüber and Adams 2001). Species that specialise in a particular feeding strategy, displaying the appropriate behaviour to capture a particular type of prey (behavioural trait) and possessing the morphological traits necessary to detect and capture that prey, reduce competition with other species (Ferry‐Graham 2002). The divergence of morphological traits associated with foraging between sympatric species to increase selectivity for particular prey types has already been described in a wide range of taxa, including the length of carnassial teeth in carnivores, beak morphology in songbirds or body size in cichlid fish (Davies et al. 2007; Winkelmann et al. 2014; Drury et al. 2018).

Variable environmental conditions act as a filter, excluding species lacking traits necessary for survival under specific conditions. This filtering promotes the emergence of traits shared among species within a community (Hoffmann and Parsons 1997). In a resource‐constrained environment, the intense selective pressure on species is expected to drive the evolution of similar adaptations to cope with harsh conditions and enable survival (Cornwell and Ackerly 2009). Such trait convergence has been reported in cave ecosystems, where the total darkness and the absence of primary producers select only species with traits enabling them to feed on detritus or by predation (Gibert and Deharveng 2002).

Limiting similarity and environmental filtering might have two opposite consequences for the structure of community traits. When competition drives limiting similarity, species traits tend to diverge, promoting high functional diversity and more efficient resource partitioning (Hooper et al. 2005). Conversely, under strong environmental filtering, species converge on similar traits, increasing functional redundancy and thus buffering against diversity loss, which in turn enhances community stability and resilience (Clarke and Warwick 1998; Hector et al. 1999; Mouillot et al. 2014). Determining whether competition or abiotic constraints predominantly shape the feeding strategies of species is therefore crucial for understanding broader ecological processes, such as energy transfer in food webs, species distributions and the evolution of competitive interactions (Holt 2009).

The oceanic pelagic ecosystem exhibits pronounced environmental gradients, from the bright, nutrient‐rich surface waters to the total darkness and scarcity of food resources found in the deeper layers, where primary production is absent (Fock et al. 2004; Sutton 2013). These unique conditions have led to the evolution of traits unique to deep‐pelagic fish communities, such as nycthemeral migration, where species migrate to the surface to feed at night and return to depth during the day, but also to morphological traits such as luminous appendages and a complex visual system (Andresen et al. 2025). It can be hypothesised that these specific traits are related, at least in part, to different feeding strategies exhibited by species along the depth gradient where resources diminish. This renders the deep‐pelagic ecosystem an ideal case for studying the assembly rules that shape the structure of traits associated with feeding strategies and resource limitation. Earlier studies analysing species traits related to feeding function in the deep‐pelagic ecosystem suggested that the low availability of food resources at depth would constrain the trophic and morphological specialisation of species (Childress and Meek 1973; Ebeling and Cailliet 1974). Contrary to this hypothesis, recent studies found significant trophic and morphological specialisation within the deep‐pelagic fish community, with low trait redundancy between species (Aneesh Kumar et al. 2017; Eduardo et al. 2020; Aparecido et al. 2023). Species living in less productive environments often possess unique biological traits, such as high resistance to starvation, low metabolic rate or exceptional foraging capacity, that can overcome the constraints associated with trophic specialisation (Francois et al. 2016; Drazen and Sutton 2017; Premate et al. 2021). Studies of this community have often focused on the mesopelagic layer, which is located approximately between 200 and 1000 m depth, where sunlight intensity is insufficient to support photosynthesis, but species can still differentiate day–night cycles. However, our understanding of how trait structure evolves in the total darkness and extreme food scarcity in the deeper layers, where species cannot rely on vertical migration to meet their energetic needs, remains deficient (Gartner et al. 2008; Drazen and Sutton 2017). Pelagic fish play an essential role in nutrient recycling and carbon transport between depth layers and serve as a significant source of prey for commercially valuable or protected species (Pusineri et al. 2005; Irigoien et al. 2014; Young et al. 2015). At the same time, deep‐pelagic fishes face increasing anthropogenic pressures, including climate change, pollution and exploitation. Thus, gaining a deeper understanding of the mechanisms shaping this community along the depth gradient is essential (Hidalgo and Browman 2019; Levin et al. 2019; Drazen et al. 2020).

A trait‐based framework is particularly valuable for investigating which mechanism primarily shapes the structure of the deep‐pelagic fish community as it offers a comprehensive approach to quantifying the role of species in a community by integrating various dimensions of their traits. This framework enables the assessment of the community vulnerability to species loss and helps identify traits that influence how communities interact with their environment (Brind'Amour et al. 2011; Mouillot et al. 2013). It also contributes to a better understanding of the evolutionary strategies that allow species to survive (Adler et al. 2014). This approach is particularly relevant because, despite its enormous biomass and the ecosystem services it provides, the deep‐pelagic fish community remains one of the least understood (Webb et al. 2010; Irigoien et al. 2014).

To understand how community trait structure varies along the resource gradient in oceanic ecosystems from epipelagic productive waters to bathypelagic dark and resource‐depleted waters, we measured morphological traits related to feeding strategies in fish species in the Bay of Biscay canyons (North‐East Atlantic). First, we tested whether limiting similarity (trait divergence) or environmental filtering (trait convergence) predominantly shapes the community structure along the depth gradient by comparing randomly generated values of functional diversity indices with the observed values. Next, we calculated the biomass‐weighted mean of traits in each depth layer to identify if certain traits respond to the environmental gradient at the community level (i.e., if mean trait values shift with depth). Finally, we investigated whether some species deviate from the predominant feeding strategy and exhibit unique combinations of traits by analysing the functional uniqueness of species in relation to their geographical restrictiveness, which helps identify potentially more vulnerable species.

## 2 Materials and Methods

### 2.1 Sampling

Species biomass data were collected at 68 stations through night‐time pelagic trawling in the canyons of the Bay of Biscay slope (North‐East Atlantic, between 43.65°N and 46.75°N of latitude and between −2.12°W and −5.17°W in longitude) during the fall between 2002 and 2022 (Table 1) (Spitz, Loutrage, Chouvelon, et al. 2024; Spitz, Loutrage, Iglesias, et al. 2024). Although changes in species distribution over time cannot be fully ruled out, a previous study of the dataset covering the period from 2002 to 2019 found no significant differences in species distribution over time (Loutrage et al. 2023). Although this result may have been influenced by the sampling design, we assumed that potential changes in the depth distribution of the 42 species studied here would not affect our main results and conclusions. Therefore, the entire data set was pooled for all analyses. This data collection occurred during the EVHOE (‘*Evaluation Halieutique de L'Ouest de l'Europe*’) scientific surveys (https://doi.org/10.18142/8), conducted by the ‘*Institut Français de Recherche pour l'Exploitation de la Mer*’ (Ifremer) aboard the R/V Thalassa. The trawl net was 192 m long with a headline of 76 m and a foot rope of 70 m. The average vertical mean mouth opening was about 24 m and the horizontal opening was about 58 m. The mesh size gradually decreases from very large 8 m (stretched mesh) at the mouth to 20 mm (stretched mesh) in the cod‐end. To allow the capture of small individuals, the trawl is also equipped with a 7.5 m long sock with a 12 mm mesh size. Midwater hauls were conducted at night from 20 to 2000 m immersion depth. Each haul was made at a specific chosen immersion depth (i.e., sampling depth). Once the trawl reached the preset depth, it was towed horizontally (i.e., constant immersion depth) for 1 h at 4 kn. A higher trawl speed on deployment and a low speed on retrieval were implemented to reduce bycatch at shallower depths than the target depth (Kashkin and Parin 1983; Sinclair et al. 1999). The term biomass refers here to the division of the biomass of each species from each trawl by the volume of water filtered during the trawl (volume filtered = vertical opening × horizontal opening × distance trawled). The resulting species biomass data by depth constitutes the community biomass matrix.

**TABLE 1:** Depth range of each depth layer and the number of trawl hauls made within each layer.

Depth layer	Depth range (m)	*n* trawl hauls	Species richness	Total biomass (kg)
Epipelagic	20–175	7	13	3.08
Upper mesopelagic	175–700	27	28	41.78
Lower mesopelagic	700–1000	16	32	40.92
Bathypelagic	1000–2000	18	42	58.84

*Note:* The number of species measured for this study and the sum of their total biomass sampled in each depth layer are also shown.

Given the unequal sampling effort across depth layers (Table 1), we cannot exclude the possibility that the reduced number of trawls in the epipelagic layer likely influenced the observed species richness and total biomass, potentially leading to an underrepresentation of rare functional traits within the epipelagic assemblage. Nevertheless, the lower species diversity in this layer aligns with recent findings from other studies on deep‐pelagic fish communities (Eduardo et al. 2022; Aparecido et al. 2023).

### 2.2 Trait Selection and Missing Data

The present study encompasses the community of deep‐pelagic fish species inhabiting the canyons of the Bay of Biscay. In this context, the term ‘deep‐pelagic’ is employed to designate small organisms inhabiting the pelagic zone at depths between 200 and 5000 m during the day (Eduardo et al. 2024). As the sampling was conducted during nocturnal hours, the presence of certain migratory species above this threshold (i.e., the epipelagic assemblage) was observed. However, from an ecological and functional perspective, these species are still considered to be part of the deep‐pelagic community. Conversely, ‘true’ epipelagic oceanic species (e.g., *Scomberesox saurus* or *Cubiceps gracilis* ) are absent from our sampling at night. The species trait dataset includes information for 42 species, comprising 722 individuals. Measurements were taken either on board during the EVHOE 2022 survey or in the laboratory, where individuals from previous sampling years were frozen prior to measurement and inclusion in the data set.

On the 42 species measured, the size range was 51.5 cm, with *Argyropelecus hemigymnus* being the smallest (3.1 ± 0.4 cm) and *Serrivomer beanii* the largest (54.6 ± 12.1 cm) (Table 2). Myctophidae and Platytroctidae were the two taxonomic families with the highest diversity of species measured (11 and 8 respectively).

**TABLE 2:** Taxonomic order and family, species depth range (m), number of individuals and standard size (cm) ± standard deviation of each species measured.

Order	Family	Species	Depth range (m)	*n* individuals	Standard length (±SD)
Myctophiformes	Myctophidae	*Benthosema glaciale*	20–2000	20	4.3 ± 0.2
		*Bolinichthys supralateralis*	735–1335	11	9.6 ± 0.6
		*Ceratoscopelus maderensis*	20–2000	30	6.3 ± 0.7
		*Diaphus metopoclampus*	735–2000	4	6.1 ± 1.7
		*Lampanyctus ater*	200–2000	22	10.9 ± 1.1
		*Lampanyctus crocodilus*	300–2000	39	10.8 ± 1.7
		*Lampanyctus macdonaldi*	1000–2000	21	12.9 ± 0.6
		*Lobianchia gemellarii*	30–2000	22	8.3 ± 0.7
		*Myctophum punctatum*	20–2000	25	6.5 ± 0.8
		*Notoscopelus bolini*	20–1335	20	7.6 ± 0.4
		*Notoscopelus kroyeri*	20–2000	36	7.6 ± 1.6
Alepocephaliformes	Platytroctidae	*Holtbyrnia anomala*	1500–1600	3	6.6 ± 0.7
		*Holtbyrnia macrops*	500–1500	9	7.9 ± 2.3
		*Maulisia argipalla*	730–1600	5	9.5 ± 1.9
		*Maulisia mauli*	685–1600	11	11.7 ± 2.8
		*Maulisia microlepis*	1000–2000	4	23.1 ± 2.2
		*Normichthys operosus*	1000–2000	38	10.5 ± 1.8
		*Sagamichthys schnakenbecki*	300–1500	9	9.2 ± 2.1
		*Searsia koefoedi*	480–2000	36	12.0 ± 1.4
	Alepocephalidae	*Photostylus pycnopterus*	1000–1600	8	9.6 ± 1.5
		*Xenodermichthys copei*	200–2000	38	11.0 ± 1.2
Stomiiformes	Stomiidae	*Borostomias antarcticus*	600–2000	14	12.2 ± 5.6
		*Chauliodus sloani*	200–2000	12	22.9 ± 5.9
		*Malacosteus niger*	510–2000	5	15.7 ± 4.1
		*Melanostomias bartonbeani*	20–2000	9	23.3 ± 2.5
		*Stomias boa*	20–2000	26	23.9 ± 4.5
	Gonostomatidae	*Cyclothone microdon*	200–2000	12	5.3 ± 0.6
		*Gonostoma elongatum*	735–1010	3	23.6 ± 1.4
		*Sigmops bathyphilus*	1000–2000	20	9.2 ± 1.8
	Sternoptychidae	*Argyropelecus hemigymnus*	150–2000	17	3.1 ± 0.4
		*Argyropelecus olfersii*	150–2000	37	5.7 ± 1.1
		*Maurolicus muelleri*	20–2000	11	3.2 ± 0.2
Aulopiformes	Paralepididae	*Arctozenus risso*	150–2000	30	15.8 ± 1.5
		*Paralepis coregonoides*	500–1600	19	7.3 ± 1.5
	Evermannellidae	*Evermannella balbo*	555–1500	4	10.5 ± 0.3
	Lestidiidae	*Lestidiops sphyrenoides*	20–1335	7	14.8 ± 0.9
Anguilliformes	Derichthyidae	*Derichthys serpentinus*	500–1600	7	22.6 ± 3.3
	Serrivomeridae	*Serrivomer beanii*	200–2000	30	54.6 ± 12.1
Argentiniformes	Bathylagidae	*Bathylagus euryops*	1335–2000	19	12.3 ± 3.6
Perciformes	Zoarcidae	*Melanostigma atlanticum*	555–1600	20	8.4 ± 1.1
Saccopharyngiformes	Eurypharyngidae	*Eurypharynx pelecanoides*	1300–2000	6	36.5 ± 6.5
Trachichthyiformes	Anoplogastridae	*Anoplogaster cornuta*	1000–1600	3	13.0 ± 1.7

A total of 26 traits were selected for their known importance in resource acquisition (Parin 1971; Gatz 1979; Webb 1984; Sibbing and Nagelkerke 2000; Karpouzi and Stergiou 2003; Dumay et al. 2004; Boyle and Horn 2006; Diderich 2006; Albouy et al. 2011; Keat‐Chuan et al. 2017; Habib et al. 2019; Andresen et al. 2025) (Table 3).

**TABLE 3:** Traits names, types, formulas or categories, description and references for the traits computed from morphological measurements.

Trait	Type	Formula/categories	Description	References
Body depth	Continuous	Body depth/standard length	Swimming capacities of fish linked to their food prospection behaviour	Diderich (2006)
Caudal throttle width	Continuous	Caudal peduncle minimum depth	Swimming strategy (cruiser/sprinter) and endurance	Albouy et al. (2011), Webb (1984)
Dorsal fin insertion	Continuous	Predorsal length/standard length	Swimming type and behaviour	Keat‐Chuan et al. (2017), Habib et al. (2019)
Eye position	Continuous	Eye height/head depth	Position in the water column (pelagic/sedentary)	Albouy et al. (2011), Gatz (1979)
Eye size	Continuous	Eye diameter/head depth	Detection of prey and visual acuity for predators	Albouy et al. (2011), Boyle and Horn (2006)
Gill outflow	Continuous	Operculum width	Suction capacity of fish	Diderich (2006)
Head length	Continuous	Head length/standard length	Maximum prey size	Habib et al. (2019)
Lower jaw length	Continuous	Lower jaw length/standard length	Compromise between power and opening speed of the mouth	Diderich (2006)
Operculum volume	Continuous	Operculum depth/operculum width	Filtering capacity and oxygen caption	Diderich (2006), Sibbing and Nagelkerke (2000)
Oral gape position	Continuous	Distance upper jaw‐bottom head/head depth	Feeding position in the water column	Albouy et al. (2011), Sibbing and Nagelkerke (2000)
Oral gape shape	Continuous	Mouth depth/mouth width	Strategy to capture prey	Albouy et al. (2011), Karpouzi and Stergiou (2003)
Oral gape surface	Continuous	Mouth width * mouth depth/body width * body depth	Type and size of prey	Albouy et al. (2011), Karpouzi and Stergiou (2003)
Orbital length	Continuous	Eye diameter/standard length	Prey size and behaviour (buried, camouflaged)	Diderich (2006), Sibbing and Nagelkerke (2000)
Pectoral fin insertion	Continuous	Prepectoral length/standard length	Manoeuvrability of fish	Keat‐Chuan et al. (2017), Habib et al. (2019)
Pectoral fin position	Continuous	Distance pectoral‐bottom body/body depth‐pectoral insertion	Pectoral fin use for manoeuvrability	Albouy et al. (2011), Dumay et al. (2004)
Transversal shape	Continuous	Body depth/body width	Position in the water column and hydrodynamism	Albouy et al. (2011), Sibbing and Nagelkerke (2000)
Gill raker type	Categorial (ordinal)	Absent or rudimentary; low‐developed; well‐developed	Filtration capacities of fish	Diderich (2006)
Oral gape axis	Categorial (ordinal)	Superior; supraterminal; terminal; subterminal; inferior	Feeding position and depth in the water column	Diderich (2006)
Chin barbel	Binary	P/A	Strategy to attract prey (lure)	Andresen et al. (2025)
Fang teeth	Binary	P/A	Enables prey to be pierced and captured	Andresen et al. (2025)
Gland head	Binary	P/A	Illumination of prey	Andresen et al. (2025)
Internal teeth	Binary	P/A	Ability to capture and handle different types and sizes of prey	This study
Large teeth	Binary	P/A	Ability to capture and handle different types and sizes of prey	Andresen et al. (2025)
Photophore position ventral	Binary	P/A	Camouflage, counter illumination	Andresen et al. (2025)
Retractable teeth	Binary	P/A	Allow species to engulf and capture large prey	Parin (1971)
Small teeth	Binary	P/A	Ability to capture and handle different types and sizes of prey	Andresen et al. (2025)

Abbreviation: P/A, presence or absence of the trait.

Twenty‐five out of 42 species (i.e., ≈60%) had at least one individual morphological measurement missing, but < 1% of morphological measurements were missing at the community level. However, as the missing data were not randomly distributed between species, an imputation algorithm was used to fill in the missing data. Removing these data could have affected our interpretation, as rare species or species from certain clades could be underrepresented (Penone et al. 2014). The approach of multivariate imputation by chained equations was therefore implemented using the R package *mice* (Van Buuren and Groothuis‐Oudshoorn 2011) with five imputations and fifty iterations. Only one species, *Eurypharynx pelecanoides* , was removed from the data set as it presented seven missing traits representing more than 25% of the data for this species.

### 2.3 Data Analyses

#### 2.3.1 Functional Space

The fish trait matrix was obtained by summarising the mean trait values of each species. To access the community's functional space, a dissimilarity matrix was first calculated from the species trait matrix using the Gower distance, as it allows both numerical and categorical variables to be included. Then, to generate the multidimensional space, a principal coordinate analysis (PCoA) was performed based on the dissimilarity matrix. The quality of the multidimensional spaces based on the PCoAs was studied as a function of the difference between the distances based on the traits and the distances in the functional space (Maire et al. 2015). A compromise between the quality of the multidimensional space and the number of axes was found at *n* = 4 (Maire et al. 2015).

#### 2.3.2 Community Structure

To investigate the functional structure and diversity of each depth layer, four diversity indices were calculated: Functional richness, which represents the proportion of functional space occupied by the species; Functional evenness, which represents the regularity of biomass distribution in functional space using the minimum spanning tree connecting all species; Functional dispersion, which represents the biomass‐weighted deviation of the species trait values from the centre of the functional space; Functional divergence, which is the proportion of biomass occupied by the species with the most extreme functional traits (Villéger et al. 2008). All functional diversity indices were calculated using the *FD* package (Laliberté et al. 2014; Laliberté and Legendre 2010).

To assess whether limiting similarity or environmental filtering shapes the structure of the deep‐pelagic fish community, we used an approach that compares observed values of each functional diversity index with values generated by a null model (Mouchet et al. 2010). To generate the null model, we randomly resampled with replacement the functional traits of each species 999 times within each depth layer. This method simulates a null model in which species traits are independent of their ecological distribution. We then calculated the four functional diversity indices based on the simulated data.

We then compared the observed values of each diversity index with the null simulated values to quantify the standardised effect size (SES) for each index, defined as:

$$\mathrm{SES}=\frac{\text{observed values}-\text{mean of simulated values}}{\text{standard deviation of simulated values}}$$

The interpretation of SES values may provide insights into the relative importance of limiting similarity versus environmental filtering in structuring the deep‐pelagic fish community. A positive SES value indicates that the functional diversity index of interest is higher than expected by chance, suggesting that limiting similarity may be the dominant process, leading to a community structure where species traits are more specialised to co‐exist or functionally diverse than randomly expected. Conversely, a negative SES value suggests that the observed functional index is lower than expected by chance, which could indicate that environmental filtering is more likely at play, resulting in a community with more similar traits and reduced functional diversity (Botta‐Dukát 2018). Values close to zero imply that the community structure aligns closely with random expectations, indicating a balance between the two mechanisms or a lack of a strong structuring mechanism. To test for significant deviation of SES values from random expectations, the distribution of simulated values of each functional diversity index measured at the sampling station and depth layer level (i.e., richness, divergence, dispersal and evenness) must be symmetric and normally distributed (Botta‐Dukát 2018). Therefore, the distribution of randomly obtained values for each index was tested for normality using the Shapiro–Wilk test, and skewness was assessed to check for symmetry. Assuming a Gaussian distribution, assemblages with SES values > 1.96 or < −1.96 are considered to significantly deviate from random expectations, indicating cases where either environmental filtering or limiting similarity plays a dominant role in shaping the community's functional composition. When the normality assumption was violated, we log‐transformed the simulated indices and used the 97.5% and 2.5% percentiles to test for significant deviations from random expectations.

#### 2.3.3 Community‐Weighted Mean

For each trait, except the two categorical traits (i.e., gill raker type and mouth opening axis), and for each sampling depth, we calculated the community‐weighted Mean (CWM), which represents the biomass‐weighted mean of the species in an assemblage. To more accurately capture the variation in trait values across the depth gradient and account for intraspecific variability, we used the set of values for each individual. This approach allowed for the recovery of the trait values for each species at specific depths, when available, rather than relying on the mean value by species. To address uncertainty, we applied nonparametric bootstrapping, as it makes no assumptions about the shape of the distribution of the population from which the sample was drawn, and resampled individuals' trait values (with replacement) in proportion to the species biomass. This generated a new set of distributions for further analysis (Maitner et al. 2023).

The community mean was weighted by species biomass rather than abundance, as biomass is directly correlated with the amount of energy and resources assimilated within a species. This is particularly relevant in the case of the deep‐pelagic community, where some species may be highly abundant but contribute only a small proportion of the total biomass. In such cases, the CWM will not accurately reflect the dominant strategies of the majority of species. However, it is important to note that species with high biomass can exert a significant influence on the CWM of an assemblage, potentially skewing the representation of community‐wide functional strategies. The R package *traitstrap* was used to perform this analysis (Maitner et al. 2023).

A principal component analysis was performed on the median of the CWM values for each depth layer to identify the dominant fish species profiles associated with different depth layers.

#### 2.3.4 Functional Rarity

To determine whether certain species were functionally rare, we calculated the functional uniqueness of each species at the community scale. Functional uniqueness measures the smallest dissimilarity between the focal species and all other species in the community. In addition, we determined the depth range restrictedness of each species, calculated as one minus the ratio of the sampling depth a species occupies over the total sampling depth. The species combining high functional uniqueness and high geographical restrictiveness were considered the most functionally rare (Violle et al. 2017). These indices were computed using the *funrar* R package (Grenié et al. 2017).

All plots were created using the *ggplot2* R package (Wickham et al. 2016), and all statistical analyses were performed in R version 4.4.2 (R Core Team 2023).

## 3 Results

### 3.1 Functional Spaces

**FIGURE 1:**
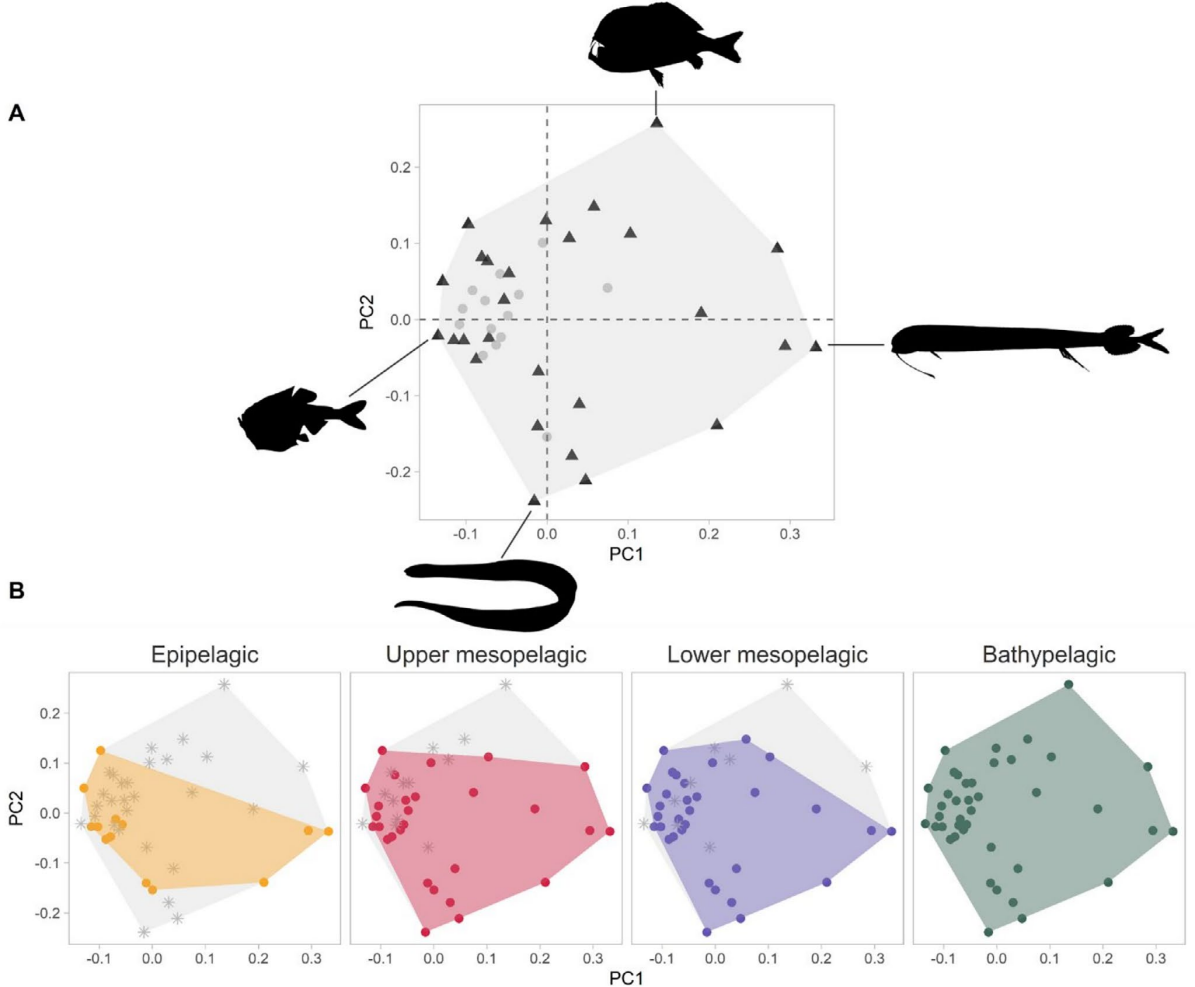
Representation of the functional space on principal coordinates 1 and 2 for the whole community (A), with vertice species represented by black triangles and nonvertice species by grey dots. A representation of the vertices species has been included to illustrate the morphological variation across the community functional space, from top to bottom: *Anoplogaster cornuta*, *Derichthys serpentinus*; from left to right: *Argyropelecus olfersii* and *Melanostomias bartonbeani*. The representation of the different depth layers (B) shows the total functional space of the community and the species absent from the depth layer in grey, while the depth functional space and the species present (dots) within it are shown in colour.

At the community level, the four‐dimensional functional space contained 26 species vertices and 15 non‐vertices (Figure 1A). The vertex species are those with the most extreme trait values and are, therefore, on the edge of the convex hull, defining its shape. At the community level, the first principal component (PCoA) explained 16% of the variation and was significantly correlated to 13 traits. The five traits that mainly explained the variance along this axis were large teeth, fang teeth, orbital length, gill raker type and head length (Eta^2^ or R^2^ value > 0.3). The PC2 accounted for 12.4% of the variance and was mainly influenced by the length of the lower jaw the gill outflow, the oral gape surface, the pectoral fin insertion and the caudal throttle width (Eta^2^ or *R* ^2^ value ≥ 0.40). The third PC axis was mainly controlled by internal teeth (Eta^2^ = 0.634), while the PC4 was influenced by the presence of ventral photophores (Eta^2^ = 0.590). Together, these four principal components explained 43.5% of the total variance.

Comparison of functional space between depth layers revealed significant similarities with a high degree of overlap between them (Figure 1B). The epipelagic zone had the smallest functional space and was therefore the least diverse. The addition of species with a rare combination of traits then expanded the functional space. The increase in the functional volume occupied by species between the epipelagic and upper mesopelagic layers was mainly due to the addition of the species *Malacosteus niger* and *Derichthys serpentinus* . The volume of functional space in the bathypelagic layer was then significantly increased by the presence of the species *Anoplogaster cornuta* .

### 3.2 Community Assembly Rules

The results of the Shapiro–Wilk test showed that only the values of the functional dispersion index followed a normal distribution without transformation (Shapiro–Wilk test, *p* > 0.05). The log transformation modified the values of the functional richness index so that they also followed a normal distribution (Shapiro–Wilk test, *p* > 0.05), and since the observed values of this index deviated from the 97.5% and 2.5% distribution percentiles of the simulated values, the SES values of this index can be interpreted as significantly deviating from the values expected by chance. Thus, these two indices, dispersion and functional richness, allow us to infer the dominance of a structuring mechanism within assemblages (environmental filtering or similarity limitation) (Botta‐Dukát 2018; De Bello et al. 2021). For the other two indices, that is, divergence and functional evenness, the values did not follow a normal distribution for the majority of depth layers, even after log transformation. However, the distribution of the simulated values of these indices was rather symmetric (only the distribution of the evenness index in the epipelagic layer was largely asymmetric, with a skewness value of 1.3), so although the significant deviation of the observed values from the null model cannot be inferred, the evolution of the values along the depth gradient can be interpreted (Öztuna et al. 2006).

**FIGURE 2:**
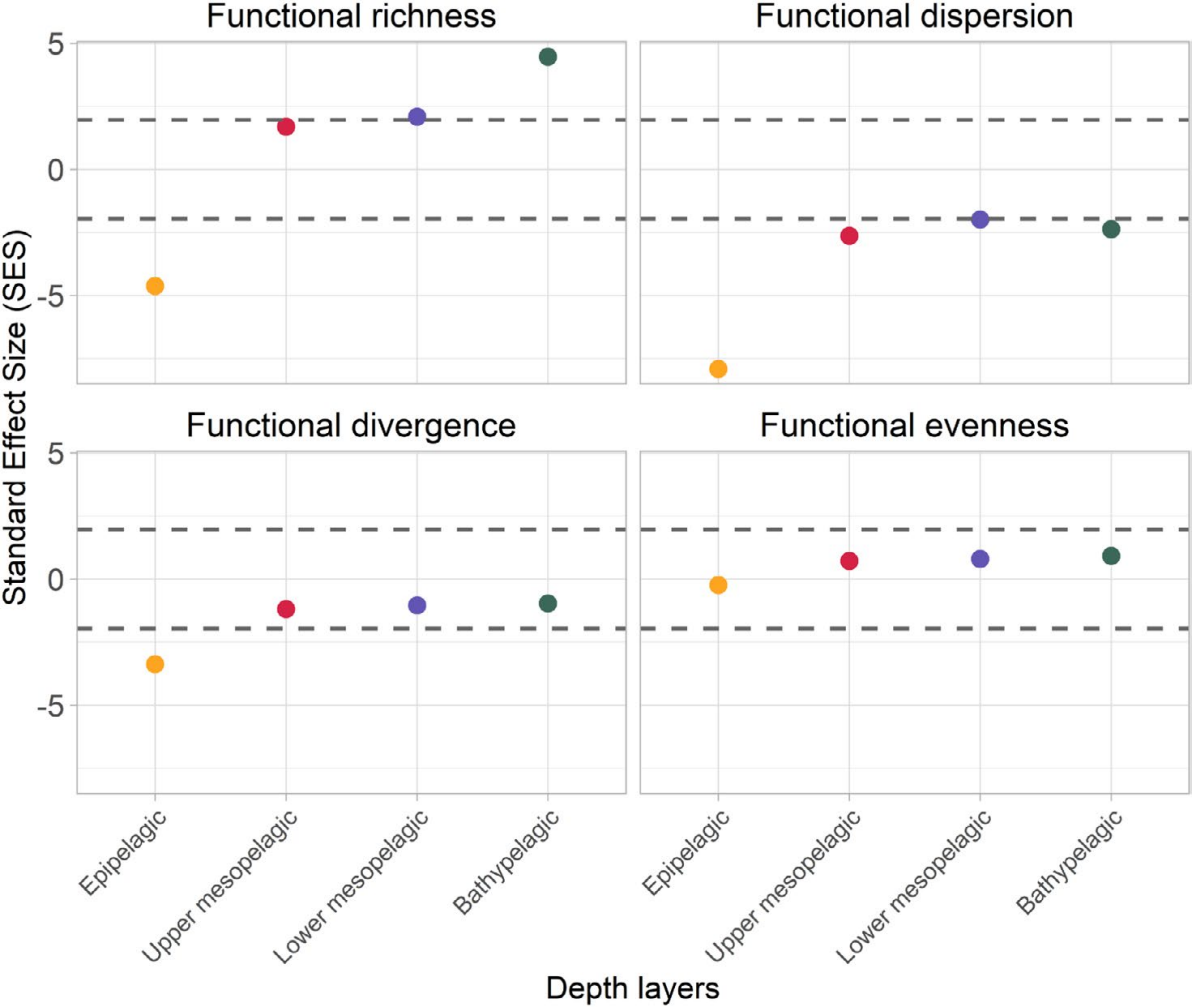
Standardised effect size (SES) values of functional richness, evenness, divergence and evenness calculated by depth layer. The epipelagic layer is represented in yellow, the upper mesopelagic layer in red, the lower mesopelagic layer in purple and the bathypelagic in green. The dashed gray lines show the level at which there is a signficant deviation from random expectations, if applicable (see the Materials and mehtods section for details).

The functional richness index showed an increase in SES values with depth, with species in the epipelagic layer exhibiting significantly lower trait diversity than expected by chance (SES functional richness values = −4.8) and, conversely, significantly higher trait diversity in the bathypelagic layer (SES functional richness values = 4.6) (Figure 2). For the dispersion index, the epipelagic layer showed a significantly negative SES value of −8.1, indicating that the dominant species in terms of biomass have similar functional traits. The upper mesopelagic and bathypelagic layers also showed negative SES values (functional dispersion SES = −2.5 and −2.6, respectively), while the lower mesopelagic layer showed values not different from those obtained at random. For the divergence index, only the epipelagic layer stood out with its low value (SES Functional Divergence = −3.4). This confirms that the species representing the highest biomass within this assemblage have convergent functional traits. Finally, the SES values of the functional evenness index showed little variability along the depth gradient.

### 3.3 Community Functional Trait Structure

**FIGURE 3:**
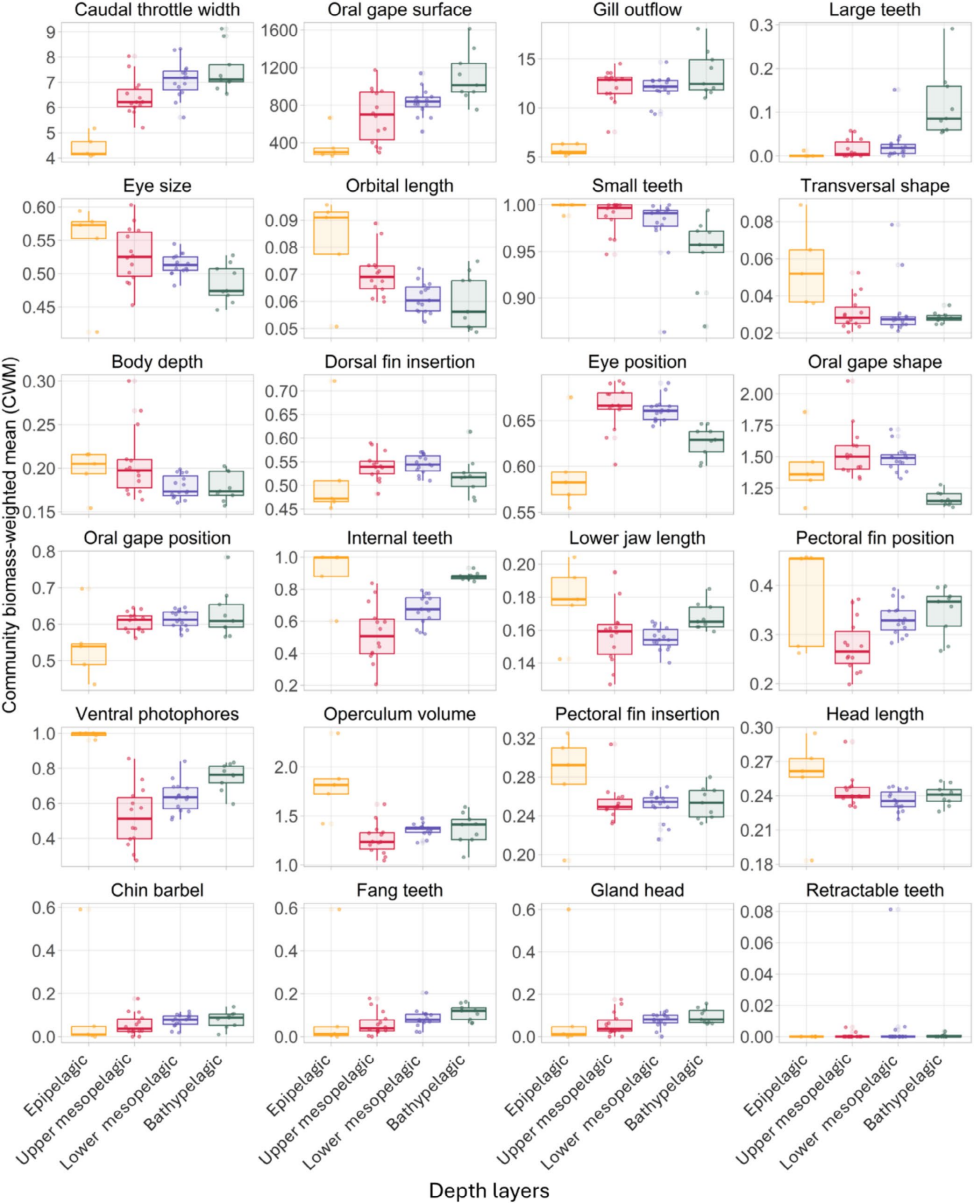
Shift in community biomass‐weighted mean (CWM) of each trait along the depth gradient. Each point represents the mean of the bootstrapped values in a sampled depth. The boxplots group the values by depth layer: the epipelagic layer in yellow, the upper mesopelagic layer in red, the lower mesopelagic layer in purple and the bathypelagic layer in green.

The CWM calculation revealed differences in value patterns along the depth gradient (Figure 3). The value of some traits increased linearly with depth; others showed extreme values in the shallower layers or maximum/minimum values at the two extremes of the depth gradient. Four traits showed increasing values with depth: caudal throttle width, oral gape surface and proportion of species with large teeth. Other traits showed decreasing values with depth, including eye size, orbital length or proportion of species with small teeth. The low value of gill outflow and the high values of operculum volume, transversal shape, pectoral fin insertion, and the high proportion of species with ventral photophores distinguished the epipelagic layer from the other depth layers. Other traits seemed to be independent of the depth gradient, such as body depth or dorsal fin insertion.

**FIGURE 4:**
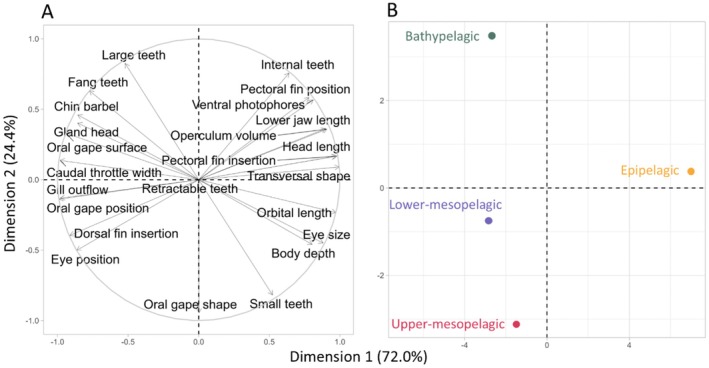
Principal component analysis (PCA) based on the median values of community biomass‐weighted mean (CWM) for each trait, with the correlation circle (A) and individual representations (B).

The PCA performed on the median CWM values per depth layer revealed distinct morphological profiles of the biomass‐dominant species in the different depth layers (Figure 4). The first dimension explained 72.0% of the variance and was strongly correlated with high values of pectoral fin insertion, transversal shape, head length, lower jaw length, operculum volume and ventral photophores and conversely with low values of caudal throttle width, oral gape position, gill outflow, and oral gape surface (Figure 4A). This combination of traits corresponds to the epipelagic species, as shown by the PCA of individuals where the epipelagic layer is the only layer with a positive value on the first dimension (Figure 4B). The three deeper layers had a similar position on this first axis and had characteristics opposite to those of the epipelagic layer.

The second dimension explained 24.4% of the variance and was mainly correlated with low values of oral gape shape and the absence of small teeth and, on the other side of the axis, with the presence of internal and large teeth. These traits were mainly supported by species from the bathypelagic layer, while species from the upper mesopelagic layer showed opposite trait values. The epipelagic and lower mesopelagic layers showed an intermediate position on this second axis.

### 3.4 Functional Rarity

**FIGURE 5:**
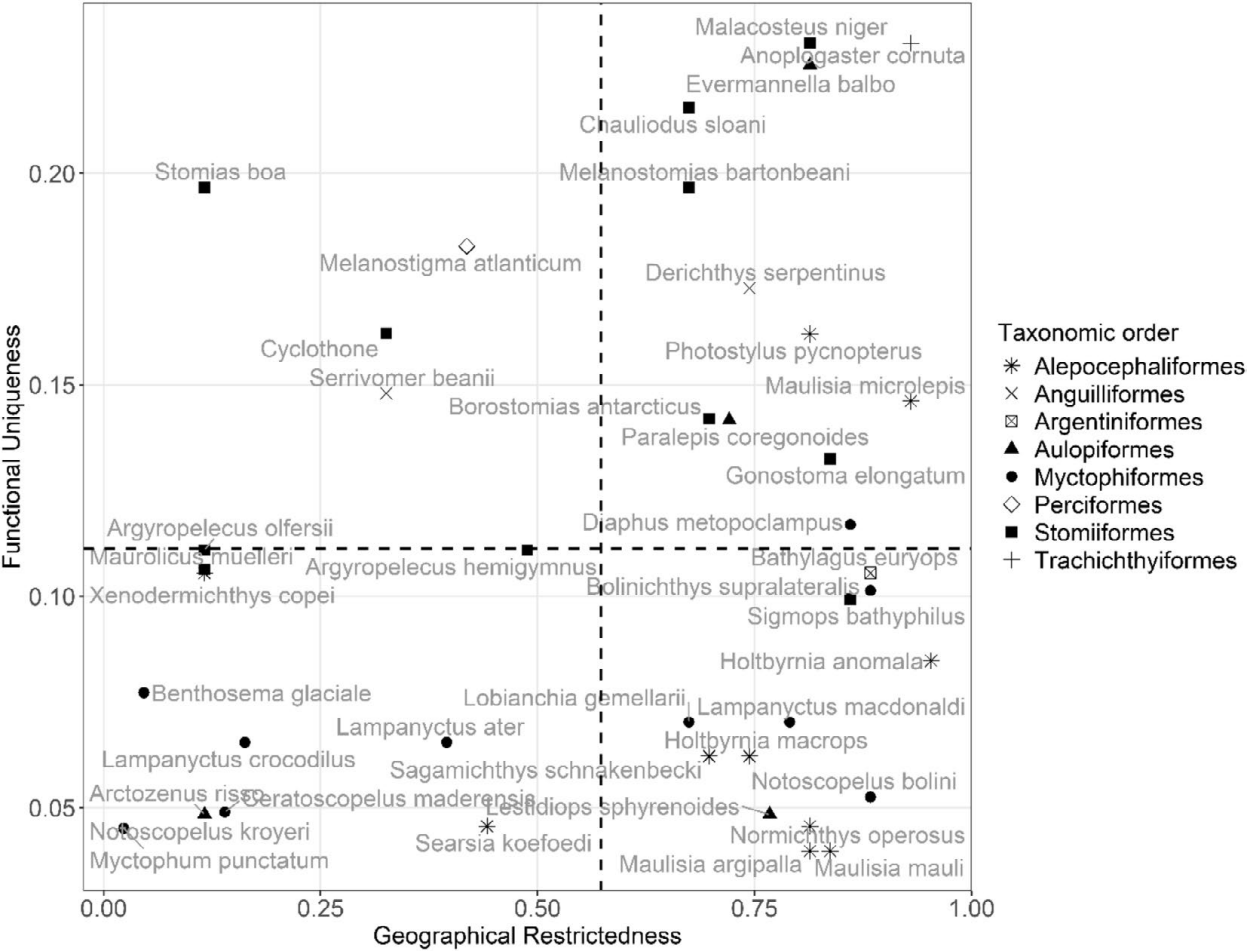
Relationship between functional uniqueness and geographical restrictiveness of each species. The dashed lines represents the median values of each index at the community level.

The species exhibiting the most common traits and, consequently, the lowest functional uniqueness values (i.e., located in the lower half of the Figure 5) were predominantly members of the most diverse families: the Myctophidae (*n* = 11 species) and the Platytroctidae (*n* = 8). Conversely, the Stomiidae, the third largest family in terms of species number, had five species with a uniqueness value exceeding 0.15, placing them among the top six most unique species. The traits that were most responsible for explaining the functional uniqueness of species were the presence of fang teeth, the absence of gill rakers or only rudimentary type, the absence of small teeth and a high pectoral fin insertion value.

When both functional uniqueness and geographical restrictiveness were considered, the Myctophidae species *Notoscopelus kroyeri* and *Myctophum punctatum* were the most frequently observed, being sampled at almost every depth and exhibiting common traits. In contrast, *Anoplogaster cornuta* , the sole representative species of the Trachichthyiformes taxonomic order, was the rarest species, displaying a unique combination of traits and being sampled exclusively in the deepest stations. The *Stomias boa* species exhibited a high degree of uniqueness and a relatively wide distribution across the depth gradient, distinguishing it from other species.

## 4 Discussion

We provided new information on the functional diversity and the assembly rules structuring the community foraging trait structure of species along a depth gradient in an understudied ecosystem, the deep‐pelagic ocean. Differences in morphological traits of the species in relation to foraging strategies were revealed along the depth gradient. While the epipelagic layer showed significant trait convergence, an increase in trait diversity as a function of depth was found, with a maximum in the bathypelagic layers, extending the result already found in studies of the mesopelagic layers (Tuset et al. 2014; Aparecido et al. 2023). The expansion of the functional space at depth was supported by rare species with unique combinations of traits that represented low biomass and had a restricted depth range distribution, making them potentially more vulnerable to disturbance. In the meantime, the limited set of strategies present within the epipelagic layer may result in a reduced capacity for diverse responses to disturbances, thereby diminishing the resilience of this assemblage.

### 4.1 Differences in Feeding Strategies Between Depth Layers

First, we found that several traits related to foraging strategy measured in our study were dependent on the depth gradient in the deep‐pelagic ecosystem, with different dominant species profiles between depth layers (e.g., oral gape surface, eye size). The important vertical gradient in light and productivity forced species to adapt to find food while limiting the risk of predation in the different environmental conditions of the deep‐pelagic ecosystem (Andresen et al. 2025). In the shallower layers, species hunt in a vision‐dominated habitat, where encounters with predators and prey are primarily mediated by vision. This has resulted in the evolution of large eyes in species within the epipelagic and upper mesopelagic assemblages (Andresen et al. 2025). This sensory system enables detection from a distance and facilitates behaviours such as stalking and pursuing prey (Childress 1985). The combination of well‐developed gill rakers, low gill outflow and high operculum volume in epipelagic species prevents the loss of small prey (Cocker 1978). It may reflect a filter‐feeding strategy that can induce feeding on small particles or prey in the manner of many small pelagic fish species in the neritic zone such as the Clupeiformes (Gerking 2014). To limit predation, almost all of these species in the illuminated layers have presented photophores in a ventral position, allowing them to mimic the descending light and reduce the risk of being spotted by predators (Lawry 1974; Denton et al. 1985).

In deeper habitats, where light is absent and food is scarce, species exhibited different trait values. Regarding direct feeding adaptations, there was an increase in the oral gape surface of species with depth. This characteristic of the deepest species was initially interpreted as a sign that these species were opportunistic feeders exploiting any potential feeding opportunities given the scarcity of food resources at depth (Childress and Meek 1973; Ebeling and Cailliet 1974). While there should be exceptions, the hypothesis of deep‐sea species feeding on a wide variety of prey types appears to have been overestimated (Drazen and Sutton 2017; Priede 2017). However, it should be noted that the current information on the diet of bathypelagic species remains insufficient and requires further investigation. Instead, the combination of large oral gape, fang‐like teeth and luminous appendages in several species may translate to a sit‐and‐wait ambush predation strategy (Drazen and Sutton 2017). Once prey is attracted by the luminous appendages, the absence of gills reduces water resistance, allowing the predator to quickly surprise and capture its prey with its large teeth, which act like a cage (Gartner Jr et al. 1997). These adaptations can increase the rate of capture per encounter and save energy dedicated to food acquisition (Borodulina 1972; Gartner Jr et al. 1997; Kenaley 2012; Drazen and Sutton 2017). Several species in our study combined these traits: *Malacosteus niger* , *Melanostomias bartonbeani* and *Chauliodus sloani* , which, although not among the species with the largest gape surface in our data set, can detach their skull and open their jaw to 90° to feed.

A study of the stomach contents of two species of stomiid fish ( *Stomias boa* and *Chauliodus sloani* ) revealed that they migrate to shallower depths in order to align with the depth at which their migrating prey can be found (Sutton and Hopkins 1996; Gartner Jr et al. 1997). They then employ a sit‐and‐wait ambush strategy to feed on them. Additionally, we observed a decrease in the transversal body profile with depth. The dominance of an elongated body morphology in deep‐sea fishes allows for a more energy‐efficient locomotor swimming style, enabling species to limit energy costs when searching for potential prey (Neat and Campbell 2013; Farré et al. 2016; Aguilar‐Medrano and Vega‐Cendejas 2020; Myers et al. 2020; Martinez et al. 2021; Gomes et al. 2024).

### 4.2 Community Assembly Rules

An increase in functional richness was observed along the depth gradient. The lowest functional diversity was observed in the epipelagic layer, which is characterised by a high concentration of food resources. While species that migrate to the surface layers may benefit from high concentrations of food resources, vertical migration is a highly energy‐demanding behaviour and increases the risk of predation, selecting species with high locomotor capabilities to hunt and avoid predation in this habitat (Seibel and Drazen 2007; Robison et al. 2020). This can result in strong selective pressure on species traits, hence the low functional richness value and high trait underdispersion observed within this layer at night. The reduction in light intensity with depth results in a decline in vision‐based interactions, particularly visual predation, leading to a decrease in selection for high locomotor capacity (Seibel and Drazen 2007). This may, in turn, result in the release of strong selection for certain species traits, thereby increasing functional diversity with depth (Webb 1984; Martinez et al. 2021). In cave ecosystems, high trophic specialisation has been found in isopod and amphipod species despite the lack of primary production (Francois et al. 2016; Premate et al. 2021). Feeding from a limited number of sources could optimise digestive costs and nutrient assimilation, which may be valuable for species in low‐productivity habitats (Britt et al. 2006; Francois et al. 2016). In the case of the deep‐pelagic ecosystem, the combination of the decrease in metabolic rate that limits energy expansion with depth and the ability of deep‐pelagic species to consume very large prey (even larger than themselves) may allow species to cope with the low resource availability at depth and allow them to specialise (Childress and Somero 1979; Seibel and Drazen 2007). In addition, the increased environmental stability at depth could further support the evolution of specialised species by providing a more predictable and constant environment (Kassen 2002; Clavel et al. 2011; Klompmaker and Finnegan 2018). This is evident in the river ecosystem, where species in the most variable habitat were structured by environmental filtering (i.e., high trait convergence). In contrast, the lower basin had more stable conditions, resulting in a trait structure shaped by resource availability, leading to high trait divergence between species (i.e., limiting similarity hypotheses) (Walsh et al. 2022). However, despite the high value of functional richness found in the bathypelagic layer (SES = 4.6), functional dispersion and divergence were low or did not deviate from randomly expected values, meaning that the dominant biomass species were not those supporting the most specialised trait combinations.

### 4.3 Functional Rarity

The presence of species with unique trait combinations has expanded the functional space and increased functional diversity at depth (e.g., *Anoplogaster cornuta, Malacosteus niger* or *Melanostomias bartonbeani* ). The inclusion of species with very specific morphologies, such as the gluper eel, *Eurypharynx pelecanoides* , which had to be removed from the analysis, would have extended the functional space even further. These species, however, typically contribute a small proportion of the total biomass and have restricted spatial distributions, representing the extreme end of the common–rare species continuum (Violle et al. 2017). Rare species in coral fish communities also exhibited a unique combination of traits (Mouillot et al. 2013). These results align with those of Coulon et al. (2023), who found that species with high geographical restrictiveness and functional distinctiveness in the North‐East Atlantic were often deep‐sea species, several of which overlap with our study. These functionally unique species may support rare ecosystem functions and enhance overall ecosystem stability. It could be argued that a community consisting of many specialist species that exhibit a high degree of complementarity in their possible responses should demonstrate greater resistance and resilience than a community consisting mainly of generalist species (Clavel et al. 2011).

The Stomiidae species present in our study exhibited high levels of functional uniqueness and are known to play a pivotal role in food webs by facilitating the transport of surface‐derived phytoplankton products from the mesopelagic zone to the bathypelagic zone and benthos (Sutton and Hopkins 1996). The Trachichthyiformes species *A. cornuta* showed the highest uniqueness value, coupled with a distinctive feeding strategy. The feeding response of this species is triggered when prey makes contact with its head region (Childress and Meek 1973). *A. cornuta* occupies a high trophic level within the deep‐pelagic fish community, with a diet derived from epipelagic sources, making it also a key player in the transfer of carbon between depth layers (Choy et al. 2015; Richards et al. 2019). However, functionally rare species are more vulnerable to demographic, environmental and genetic stochasticity due to their rarity (Simberloff 1986; Nogueira et al. 2010; Auber et al. 2022). Despite their unique and often irreplaceable ecological roles, these species are likely to be the first to go extinct in the face of the crisis of biodiversity loss (Bracken and Low 2012; Leitão et al. 2016; Coulon et al. 2023).

While species from the families Myctophidae and Platytroctidae had the lowest functional uniqueness values, their nycthemeral migration and potential benthopelagic feeding behaviours contribute distinctly to food webs by facilitating the transport and distribution of organic matter across trophic levels (Aguilar‐Medrano and Vega‐Cendejas 2020). The ability of communities to respond in various ways is crucial for mitigating the consequences of global change (Loreau and Hector 2001; Leitão et al. 2016). Despite currently being listed as Least Concern on the IUCN Red List, there is a significant lack of data on deep‐pelagic fish species, including their population dynamics, geographic distribution and life history parameters such as age at maturity and reproductive rate. This information gap hampers our ability to fully assess the vulnerability of these species and their potential for decline. This is particularly concerning in the deep sea, where human pressures like fishing are, for now, less pronounced, but where species have specialised foraging strategies in the relatively stable environmental conditions, making them more vulnerable to change (Clavel et al. 2011). The presence of many functionally rare species may also reflect an ecosystem with near‐pristine conditions, where human impacts have been less pronounced compared to exploited neritic ecosystems where high fishing pressure has reduced the presence of functionally rare species (Murgier et al. 2021). The characteristics observed in the deep‐sea could provide information about the functional structuring of near‐pristine fish communities. These observations highlight the importance of integrating a trait‐based approach into conservation policies to identify hotspots of community diversity, particularly for species with insufficient data (Luiz et al. 2016; Grenié et al. 2018; Walls and Dulvy 2020).

The difficulty of accessing deep‐sea species is an important consideration when interpreting functional roles from their morphological traits. Few in situ observations have been made, and there is still a significant knowledge gap about deep‐sea fish ecology, life history and metabolism, limiting our understanding of how the traits of species are linked to their functional roles. The framework of trait–function relationships developed for shallow‐water species may not fully apply to deep‐sea species. An interesting illustration is the species *Malacosteus niger* , which, as described above, has morphological adaptations for capturing large prey (i.e., large gape, no gill raker and large teeth). However, studies of the diet of this species in several oceanic basins have shown that it is predominantly a zooplanktivore, which is different from other stomiidae species (Sutton 2005). Coupling species traits with dietary information is essential for enhancing our understanding of the functional ecology of the diverse species in extreme environments. Pursuing the trait‐based approach in these challenging ecosystems should expand the boundaries of our scientific knowledge and explore mechanisms beyond our current imagination.

We hope that our study may contribute to the debate on possible management implications for these ecosystems. Our findings suggest that conservation efforts should protect habitat heterogeneity, including full depth gradients in marine protected areas, and ensure connectivity for species that migrate vertically. Due to current knowledge limitations, it is essential to address critical data gaps through targeted research and international data sharing, alongside precautionary management. Functional trait indicators should be incorporated into long‐term monitoring programmes to effectively detect early changes to the ecosystem and track community resilience.

## Author Contributions

**Liz Loutrage:** conceptualization (equal), formal analysis (equal), investigation (equal), writing – original draft (equal). **Anik Brind'Amour:** conceptualization (equal), formal analysis (equal), funding acquisition (equal), writing – original draft (equal). **Benoit Simon‐Bouhet:** formal analysis (supporting), writing – review and editing (equal). **Rachel Dubourg:** investigation (supporting), writing – review and editing (equal). **Célina Chantre:** investigation (equal). **Jérôme Spitz:** conceptualization (equal), formal analysis (supporting), funding acquisition (equal), investigation (equal), resources (equal), writing – original draft (equal).

## Conflicts of Interest

The authors declare no conflicts of interest.

## Supporting information


**Appendix S1:** ece371891‐sup‐0001‐AppendixS1.docx.

## Data Availability

The raw morphological data are provided data. InDoRES platform https://doi.org/10.48579/PRO/FNVH6Z. The trawl data sets (including metadata) with species biomass data (2002–2019) are available on the PANGAEA platform https://doi.pangaea.de/10.1594/PANGAEA.967132 and the data. InDoRES platform (2021–2022) https://doi.org/10.48579/PRO/AIKOEB. The code to reproduce the full analysis is provided on GitHub https://github.com/lizloutrage/functional_diversity.
