# Supplementary material for: Unlocking the Depths: Use of a Trait‐Based Approach to Reveal the Diversity of Foraging Strategies in a Deep‐Pelagic Fish Community
Source: Ecol Evol. 2025 Jul 28;15(8):e71891. doi: 10.1002/ece3.71891 (PMC12304439; doi:10.1002/ece3.71891)
Supplement: Supplementary file 1 — Appendix S1: ece371891‐sup‐0001‐AppendixS1.docx. [file ECE3-15-e71891-s001.docx]

**Supporting information**

**
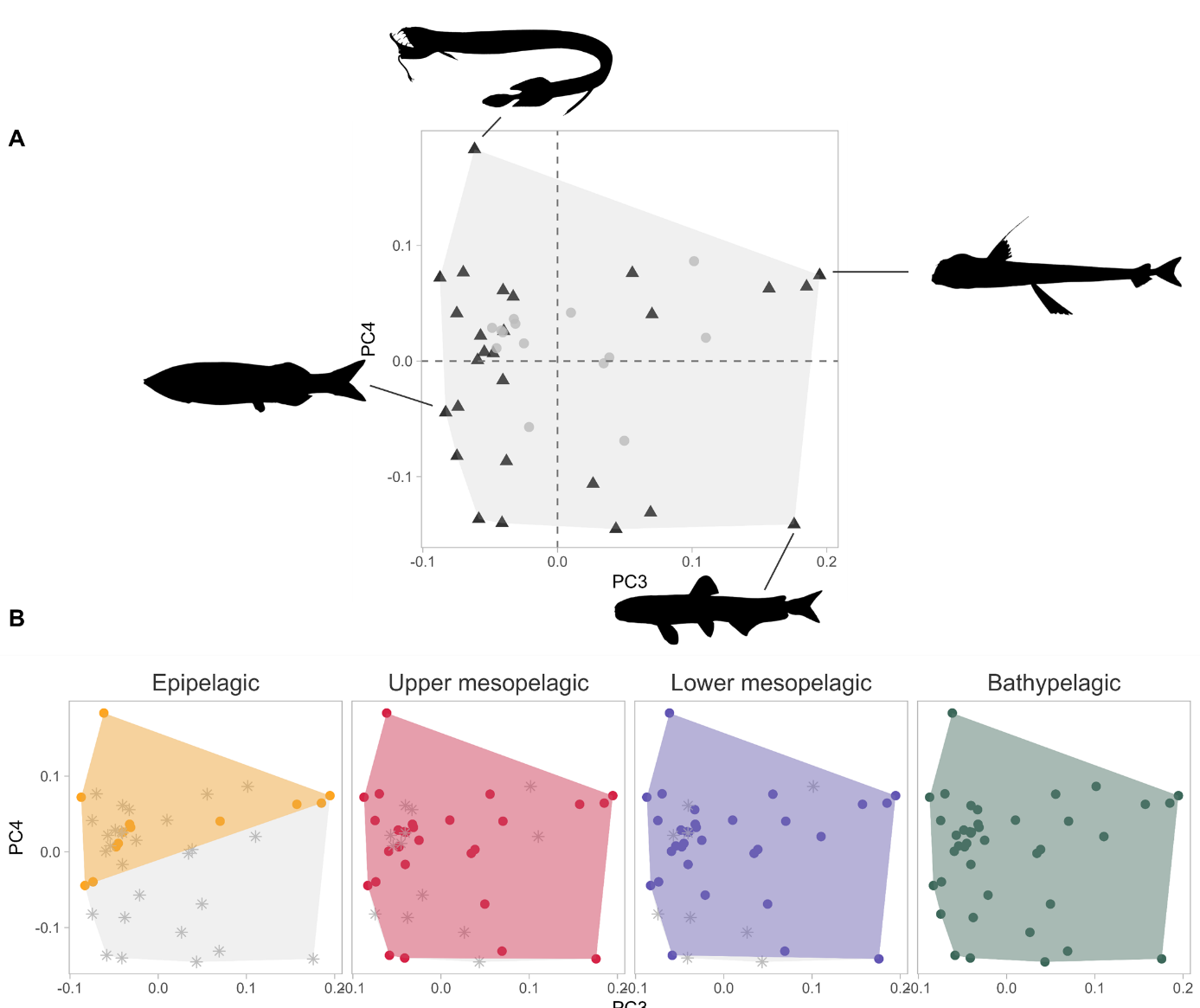
Appendix 1.** Representation of functional space on principal cooordinates 3 and 4 for the total community (A) with the vertices species represented in black triangles. A representation of the species vertices has been included to illustrate the morphological variation across the community functional space, from top to bottom: Stomias boa, Evermannella balbo and from left to right: Maulisia microlepis and Chauliodus sloani. The representation of the different depth layers (B) shows the total community functional space and the species absent from the depth layer as grey, while the species present in the depth layers are shown in colours.
